# On-farm multi-location evaluation of genotype by environment interactions for seed yield and cooking time in common bean

**DOI:** 10.1038/s41598-020-60087-2

**Published:** 2020-02-27

**Authors:** Dennis N. Katuuramu, Gabriel B. Luyima, Stanley T. Nkalubo, Jason A. Wiesinger, James D. Kelly, Karen A. Cichy

**Affiliations:** 1 Department of Plant, Soil and Microbial Sciences, Michigan State University, East Lansing, MI 48824 USA; 2 Legumes Research Program, National Crops Resources Research Institute, Namulonge, Kampala Uganda; 3 Present Address: USDA-ARS, Robert W. Holley Center for Agriculture and Health, Cornell University, Ithaca, NY 14853 USA; 4 USDA-ARS, Sugarbeet and Bean Research Unit, Michigan State University, East Lansing, MI 48824 USA

**Keywords:** Genetics, Plant sciences

## Abstract

Common bean variety choice by farmers in Uganda is driven by seed yield plus end-use quality traits like market class and cooking time. Limited genotype by environment information is available for traits valued by consumers. This research evaluated yield, seed size, hydration properties, and cooking time of 15 common bean genotypes within market classes recognized by consumers along with three farmers’ checks at nine on-farm locations in Uganda for two seasons. Yield ranged from 71 to 3,216 kg ha^−1^ and was largely controlled by location (21.5% of Total Sums of Squares [TSS]), plus the interaction between location and season (48.6% of TSS). Cooking time varied from 19 to 271 minutes with the genotypes Cebo Cela and Ervilha consistently cooking fastest in 24 and 27 minutes respectively. Comparatively, the local checks (NABE-4, NABE-15, and Masindi yellow) took 35 to 45 minutes to cook. Cooking time was largely controlled by genotype (40.6% of TSS). A GGE biplot analysis uncovered the presence of two mega-environments for yield and one mega-environment for cooking time. Identification of mega-environments for these traits will help expedite common bean breeding, evaluation, and variety selection through reduction of number of test environments needed for phenotype evaluations. The high yielding and fast cooking genotypes from this study can be targeted as parental materials to improve existing common bean germplasm for these important traits.

## Introduction

The world population is projected to reach 10 billion by the year 2050 and crop varieties that are resilient, productive, and nutritious under changing biotic and abiotic threats are urgently needed to meet the food security needs of the expanding population^1^. Common bean (*Phaseolus vulgaris* L.) is a widely grown and consumed staple crop in many parts of Latin America and Africa including Uganda^2,3^. In Eastern Africa, Uganda is the second largest producer of common bean and consumption provides 25% of the calories and 45% of the daily protein requirement^4^. In Uganda, on-farm yields are often very low compared to those reported on research farms^5^. Reasons for the low on-farm yields include: (1) Use of poor-quality farmer saved seed; (2) Use of unimproved landrace genotypes that are susceptible to diseases and insect pests; (3) The poor soil fertility conditions and variable weather patterns in the production areas^6^. In Uganda and throughout Eastern Africa, farmers often place a greater value on end-use characteristics like seed color, seed size, flavor, and cooking time when compared to actual seed yield^7^.

Determining the degree of genotype by environment interactions for yield performance in common bean has been a major focus for plant breeders^8^. Seed yield performance is often measured through collaboration at University research sites, International Center for Tropical Agriculture and the National Agricultural Research System. However, there is limited information on studies conducted on farms owned and managed by the common bean growers^8^. On-farm participatory evaluation of genotype and genotype by environment interactions can identify novel germplasm suited for specific agro-ecological conditions, as previously demonstrated for rice in India^9^, sweet potatoes in Uganda and South Africa^10,11^, and cassava in Tanzania^12^. Genotypic yield performance is influenced by multiple genes interacting with biotic and abiotic stress factors over the course of the crop’s growing season^13^. Additionally, evaluation of germplasm on farmers’ fields enables plant breeders to determine crop genotype performance amidst natural disease and pest pressures in the target locations^14^.

Recent studies in Uganda have revealed that common bean farmers attach equal value to both agronomic performance and end-use quality traits^15,16^. Therefore, new common bean varieties for Uganda must have improvements for both seed yield and end-use quality traits. One important end-use quality trait in common bean is cooking time because the crop often requires large amounts of heat energy to cook before consumption^17^. A large proportion of the population in Eastern Africa use either fuelwood or charcoal as the primary source of energy for cooking^18^. The process of gathering (and purchasing) wood fuel for cooking is a labor-intensive and time-consuming activity for families especially those living in the rural communities^19^. Genetic variability for cooking time exists in common bean and there is potential to improve this trait in new varieties belonging to the market classes recognized by the consumers^20,21^.

Cooking time has been shown to be highly heritable with limited effects of genotype by environment interactions^22,23^. On-farm trials that include cooking time evaluation offer numerous advantages, including direct feedback about the harvested materials from the end users. Additionally, the approach allows for measurement of genotype performances at the actual environments where the future common bean varieties will be released for adoption, production, consumption, and marketing^24^.

Given common bean’s wide range adaptation and importance as a food and nutrition security crop, our study evaluated the magnitude of genotype plus genotype by environment responses for seed yield, seed size, water uptake, and cooking time traits in 15 common bean test genotypes across nine on-farm locations spanning three agro-ecological zones in Uganda. The test genotypes were evaluated along with the farmers’ local check varieties. Local check varieties were included to serve as benchmarks for each measured trait and determine the overall potential value of the test genotypes as parental materials to improve common bean for the aforementioned traits.

## Results

### ANOVA and broad-sense heritability

Results of the analysis of variance (ANOVA) for all traits are presented in Table 1.

**Table 1:** ANOVA showing mean squares and percentage of total variance explained for yield, seed weight, water uptake, and cooking time of the common bean genotypes evaluated for two field seasons at the nine on-farm locations in Uganda.

Source of variation	df	Traits							
		Yield (kg ha^−1^)		Seed weight (g)		Water uptake (%)		Cooking time (min)	
		Mean square	% TSS	Mean square	% TSS	Mean Square	% TSS	Mean square	% TSS
G	15	558104.2**	4.8	1351.4**	51.5	5516.3**	45.4	12850.9**	40.6
L	8	4738947.6**	21.5	327.5**	6.7	548.2**	2.4	4502.0**	7.6
S	1	719139.3**	0.4	515.1**	1.3	1906.1**	1.0	16324.3**	3.4
GxL	120	100884.9**	6.9	16.0*	4.9	150.1**	9.9	763.0**	19.3
GxS	15	169218.6**	1.4	48.0**	1.8	2997.9**	24.7	3606.8**	11.4
LxS	8	10706389.0**	48.6	981.6**	19.9	907.8**	4.0	665.5**	1.1
rep(LxS)	18	146755.7**	1.5	16.2 NS	0.7	47.8*	0.5	55.3 NS	0.2
GxLxS	120	100468.7**	6.8	18.6**	5.7	136.3**	9.0	549.6**	13.9

df: degrees of freedom; Significance level: *P value < 0.01; **P value < 0.001; NS = not significant; % TSS: Percentage of TSS explained.

### Agronomic traits

The most important source of variation for seed yield was location (21.5% of total sums of squares [TSS]) and the interaction component between location and season (48.6% of TSS) (Table 1). Seed weight was largely controlled by genotype (51.5% of TSS) followed by location by season interaction (19.9% TSS) (Table 1). Broad-sense heritability (H^2^) estimates for all traits ranged from 46.8 to 96.5%. Seed yield had a moderate heritability estimate of 69.7% while seed weight had a high broad-sense heritability estimate of 96.5%.

### Hydration properties and cooking time

The ANOVA for water uptake showed that genotype and the interaction term between genotype and season were the major sources of variation as they accounted for 45.4 and 24.7% of TSS respectively (Table 1). Cooking time was controlled to a large extent by genotype (40.6% of TSS) followed by genotype by location (19.3% of TSS) (Table 1). Cooking time had a moderately high broad sense heritability estimate of 70.3%.

## Evaluation of Genotype and Environment Performances

### Agronomic traits

Seed yield among genotypes ranged from 71 to 3,216 kg ha^−1^ (Supplementary Table S2). The highest seed yield was recorded for genotypes G8 (Chijar) from the Caribbean and G5 (Amarelo Cela) - a landrace from sub-Saharan Africa (Table 2). The two highest yielding genotypes had an indeterminate growth habit. The third highest yielding genotype G3 (PI527538), is a yellow landrace also from sub-Saharan Africa with a determinate growth habit. Genotypes G12 (Uyole 96), G4 (Cebo Cela) and G13 (Charlevoix) produced significantly lower yields (Table 2). All the local check genotypes (NABE-15, NABE-4, and Masindi yellow) had yields lower than the top three genotypes of Chijar, Amarelo Cela, and PI527538 (Table 2).

**Table 2:** Genotype means for the observed traits of the common bean genotypes evaluated across nine field sites for two years in Uganda.

Genotype code	Genotype name	Yield (kg ha^−1^)	Seed weight (g)	Water uptake (%)	Cooking time (min)
G1	Blanco Fanesquero	593	45.3	111.5	28.2
G2	Ervilha	671	42.2	119.3	27.0
G3	PI527538	786	40.1	106.8	43.9
G4	Cebo Cela	531	35.2	114.6	24.6
G5	Amarelo Cela	910	26.6	71.2	96.5
G6	Maalasa	628	40.1	113.0	35.8
G7	Rozi Koko	589	42.4	114.9	32.8
G8	Chijar	929	25.0	99.8	53.8
G9	Vazon 7	645	30.4	90.4	65.0
G10	PR0737-1	709	34.2	110.7	72.4
G11	Kidungu	561	34.1	101.1	44.8
G12	Uyole 96	525	44.7	107.3	41.3
G13	Charlevoix	536	41.6	122.1	48.9
G14	Selian 97	714	35.2	113.9	33.1
G15	Sacramento	587	40.3	113.1	43.1
G16	NABE-15	735	40.5	103.7	35.7
Check-2	NABE-4	472	44.7	108.6	38.2
Check-3	Masindi yellow	439	37.6	115.4	45.6
**LSD (α** = **0.05)**		**107**	**1.5**	**2.1**	**3.0**

LSD: Least significant difference used to compare genotype performances for the measured traits; G16 denotes the NABE-15 local check-1 variety that was grown at locations KA, TU, KY, GE, KV, and BA. NABE-4 was evaluated at location AG while Masindi yellow was grown at locations KU and TW.

Differences in environmental mean performance were large for yield with values ranging from 336 to 1,057 kg ha^−1^. Location KV in Rakai district had the highest yield performance followed by locations KA and TU from Hoima district (Table 3). Seed yield was lowest at TW and KU locations both from Kamuli district. These locations had drier growing seasons and also had high disease pressure especially from common bean bacterial blight (*Xanthomonas axonopodis* pv. *phaseoli*) and bean common mosaic virus (a member of the Potyvirus).

**Table 3:** Environmental means for the measured traits across the common bean genotypes evaluated at nine on-farm field sites for two years in Uganda.

Location code	Yield (kg ha^−1^)	Seed weight (g)	Water uptake (%)	Cooking time (min)
KA	897	37.6	111.9	43.0
KY	695	34.4	110.7	50.6
TU	981	35.4	109.2	44.2
KU	350	34.8	105.8	38.6
TW	336	37.1	103.7	65.9
GE	666	38.4	103.4	42.4
AG	418	39.2	107.9	44.9
KV	1057	41.5	106.2	39.5
BA	637	37.9	106.9	41.1
**LSD (α** = **0.05)**	**80**	**1.2**	**1.6**	**2.3**

LSD: Least significant difference used to compare environmental performances for the measured traits.

Seed weight varied from 19–63 g per 100 seeds (Supplementary Table S3). The highest seed weight was observed among genotypes G1 (Blanco Fanesquero), G12 (Uyole 96), NABE-4, and G7 (Rozi Koko). The lowest seed weight values were observed among genotypes G8 (Chijar), G5 (Amarelo Cela), and G9 (Vazon 7) (Table 2). All the genotypes with the highest seed weight mean values (Blanco Fanesquero, Uyole 96, Rozi Koko, and Ervilha) had a determinate growth habit. The smallest seed weight values were recorded among genotypes Chijar, Amarelo Cela, and Vazon 7 which had an indeterminate growth habit (Tables 2 and 4). The local check genotypes had seed sizes ranging from 37.6 to 44.7 g per 100 seeds (Table 2). Seed weight was highest among genotypes grown at locations KV and AG in Rakai district (Table 3). The smallest seeds were produced at locations KY and KU from Hoima and Kamuli district respectively (Table 3).

**Table 4:** Description of the experimental common bean genotypes evaluated over two years across nine on-farm locations in Uganda.

Genotype code	Genotype name	Gene pool	Region of origin	Country of origin	Cultivation status	Seed type	Growth habit
G1	Blanco Fanesquero	Andean	South America	Ecuador	Variety	White	Determinate
G2	Ervilha	Andean	Southern Africa	Angola	Landrace	Yellow	Determinate
G3	PI527538	Andean	East Africa	Burundi	Landrace	Yellow	Determinate
G4	Cebo Cela	Andean	Southern Africa	Angola	Landrace	Yellow	Indeterminate
G5	Amarelo Cela	MA	Southern Africa	Angola	Landrace	Yellow	Indeterminate
G6	Maalasa	Andean	East Africa	Tanzania	Landrace	Red mottled	Determinate
G7	Rozi Koko	Andean	East Africa	Tanzania	Landrace	Red mottled	Determinate
G8	Chijar	MA	Caribbean	Puerto Rico	Landrace	Red mottled	Indeterminate
G9	Vazon 7	MA	Caribbean	Puerto Rico	Landrace	Red mottled	Indeterminate
G10	PR0737-1	Admix	Caribbean	Puerto Rico	Variety	Red mottled	Indeterminate
G11	Kidungu	Andean	East Africa	Tanzania	Landrace	Small red	Determinate
G12	Uyole 96	Andean	East Africa	Tanzania	Variety	DRK	Determinate
G13	Charlevoix	Andean	North America	U.S.	Variety	DRK	Determinate
G14	Selian 97	Andean	East Africa	Tanzania	Variety	DRK	Determinate
G15	Sacramento	Andean	North America	U.S.	Variety	LRK	Determinate
**G16 -Local Checks**							
Check-1	NABE-15	Andean	East Africa	Uganda	Variety	Cream mottled	Determinate
Check-2	NABE-4	Andean	East Africa	Uganda	Variety	Red mottled	Determinate
Check-3	Masindi yellow	Andean	East Africa	Uganda	Landrace	Yellow	Determinate

*MA*: Middle American; *DRK:* Dark red kidney; *LRK:* Light red kidney.

### Water uptake and cooking time

Seed water uptake after soaking varied from 18.7 to 149.1% (Supplementary Table S4). Genotypes G13 (Charlevoix) and G2 (Ervilha) absorbed the most water while accessions G5 (Amarelo Cela) and G9 (Vazon 7) absorbed the least amount of water (Table 2). The genotypes grown at locations KA and KY in the Hoima district imbibed the most water (Table 3). Genotypes harvested from locations GE and TW in Kamuli district had the lowest water absorption (Table 3).

Cooking time among genotypes varied from 19.4 to 270.6 minutes (Supplementary Table S5). Genotypes with the longest cooking times were G5 (Amarelo Cela), G10 (PR0737-1), and G9 (Vazon 7) (Table 2). The longest cooking genotype Amarelo Cela was a yellow bean landrace collected from Angola (Table 2). Amarelo Cela (G5) and Vazon 7 (G9) also absorbed the least amount of water during soaking and the low water uptake likely contributed to the longer cooking times observed for these genotypes. The fastest cooking genotypes G4 (Cebo Cela) and G2 (Ervilha) are both African Manteca yellow common bean landraces collected from Angola and G1 (Blanco Fanesquero) is a white grain colored variety from Ecuador (Table 2). The local check genotypes had average cooking times ranging from 35.7 to 45.6 minutes (Table 2). Cooking time averages varied across locations. Genotypes grown at location TW in Kamuli district required the longest time to cook (65.9 minutes) while genotypes grown at sites KU and KV had the shortest cooking times of 38.6 and 39.5 minutes respectively (Table 3).

## Polygon (“which-won-where”) View of the GGE Biplots

### Seed yield and seed weight

**Figure 1:**
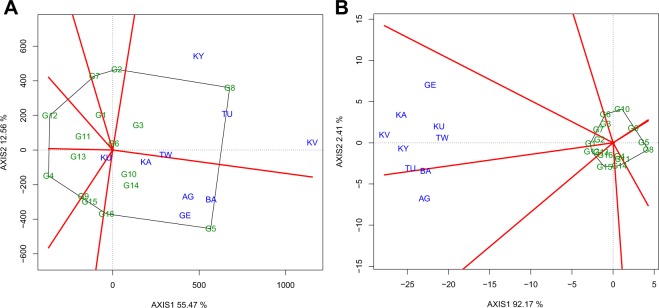
The polygon (which-won-where) view of genotype main effects plus genotype by environment interaction effect (GGE) biplot of the common bean genotypes evaluated for two years across nine on-farm locations for yield (**A**) and seed weight (**B**). The biplots were generated based on a Scaling = 0, Centering = 2, and SVP = 2. Key to the labels of locations and genotypes is presented in Tables 1 and 2 respectively.

The polygon view of the GGE biplot explained 68 and 94.6% of the genotype plus genotype by environment variation for seed yield and seed weight respectively (Fig. 1: Panels A and B). The GGE biplot analysis for yield resulted in two sectors indicating presence of two winning genotypes of G8 (Chijar) and G5 (Amarelo) for each sector. The presence of two sectors also confirms presence of genotype by environment interaction and two mega-environments for seed yield (Fig. 1A). The first mega-environment had locations KY, TU, KV, and TW while sites KA, AG, BA, GE, and KU formed the second mega-environment (Fig. 1A). The test environments clustered in two groups for seed weight implying presence of two mega-environments (Fig. 1B). The first mega-environment for seed weight was comprised of locations GE, KA, KV, KU, TW, KY, and TU. The second mega-environment had locations BA and AG (Fig. 1B).

### Water uptake and cooking time

**Figure 2:**
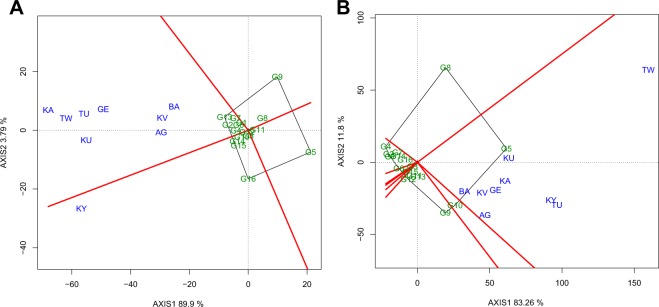
The polygon (which-won-where) view of genotype main effects plus genotype by environment interaction effect (GGE) biplot of the common bean genotypes evaluated for two years across nine on-farm locations for water uptake (**A**) and cooking time (**B**). The biplots were generated based on a Scaling = 0, Centering = 2, and SVP = 2. Key to the labels of locations and genotypes is presented in Tables 1 and 2 respectively.

The GGE biplot explained 93.7 and 95.1% of the genotype plus genotype by environment variation for water uptake and cooking time respectively (Fig. 2: Panels A and B). The locations clustered into two sectors for water uptake indicating the presence of two mega-environments (Fig. 2A). Eight of the nine locations made one mega-environment (Fig. 2A). All the environments clustered in one sector (one mega-environment) for cooking time with one clear slow-cooking genotype G5 (Amarelo Cela) and the fastest cooking genotype was G4 (Cebo Cela) (Fig. 2B).

## Genotype Mean Performance vs. Stability GGE Biplots

### Seed yield and seed weight performance

**Figure 3:**
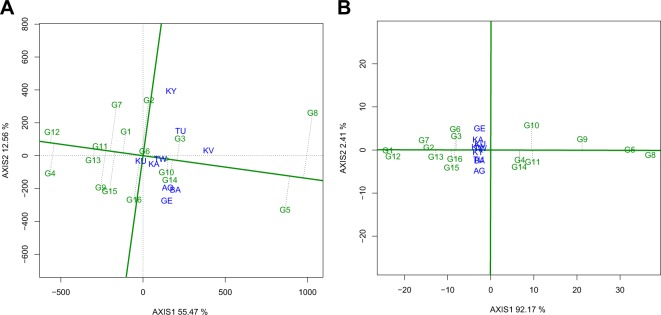
Mean performance vs. stability view of genotype main effects plus genotype by environment interaction effect (GGE) biplot of the common bean genotypes evaluated for two years across nine on-farm locations for yield (**A**) and seed weight (**B**). The biplots were generated based on a Scaling = 0, Centering = 2, and SVP = 1. Key to the labels of locations and genotypes is presented in Tables 1 and 2 respectively.

Genotypes trending towards the direction of G5 (Amarelo Cela) and G8 (Chijar) denote higher yielding and genotypes trending towards the opposite direction represent the poor performing lines like G4 (Cebo Cela) and G12 (Uyole 96) (Fig. 3A). Genotypes G6 (Maalasa) and G11 (Kidungu) were the most stable as they had near zero projection from the AEC horizontal axis. Genotypes G8 (Chijar) and G2 (Ervilha) are regarded as the least stable for yield performance since they exhibited the longest projection from the AEC horizontal axis (Fig. 3A). Genotypes in the direction of G1 (Blanco Fanesquero) and G12 (Uyole 96) had a larger seed size while genotypes in the direction of G5 (Amarelo Cela) and G8 (Chijar) had a smaller seed size (Fig. 3B). Genotypes G1 (Blanco Fanesquero), G2 (Ervilha), and G5 (Amarelo Cela) were the most stable for seed weight as they had a near zero projection from the AEC horizontal axis. The least stable genotypes for seed weight were G6 (Maalasa) and G10 (PR0737-1) as these exhibited the longest projections from the AEC horizontal axis (Fig. 3B). Genotypic performance instability (longer projections) can also indicate local adaptation, whereby genotypes located above or below the AEC horizontal axis would perform better at test environments located in identical orientations of the AEC axis.

### Water uptake and cooking time

**Figure 4:**
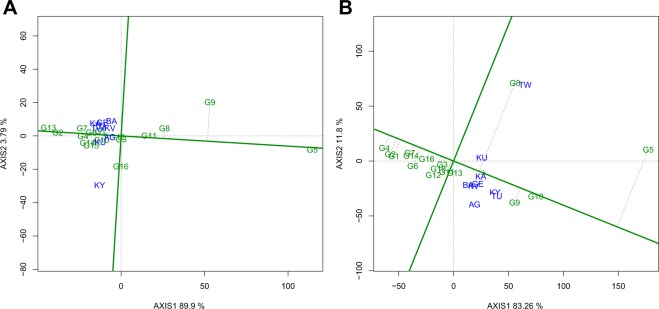
Mean performance vs. stability view of genotype main effects plus genotype by environment interaction effect (GGE) biplot of the common bean genotypes evaluated for two years across nine on-farm locations for water uptake (**A**) and cooking time (**B**). The biplots were generated based on a Scaling = 0, Centering = 2, and SVP = 1. Key to the labels of locations and genotypes is presented in Tables 1 and 2 respectively.

Genotypes in the direction of G2 (Ervilha) and G13 (Charlevoix) absorbed more water while genotypes in the direction of G9 (Vazon 7) and G5 (Amarelo Cela) had lower water uptake values (Fig. 4A). Most genotypes were stable for water uptake across locations except for G9 (Vazon 7) which had a long projection from the AEC horizontal axis (Fig. 4A). Genotypes in the direction of G10 (PR0737-1) and G5 (Amarelo Cela) were slow cooking while genotypes in the opposite direction along the AEC horizontal axis such as G1 (Blanco Fanesquero), G2 (Ervilha), and G4 (Cebo Cela) were fast cooking. The Manteca yellow bean Cebo Cela was the fastest cooking genotype in this study (Fig. 4B). Genotype G10 (PR0737-1) was the most stable for cooking time (with a near zero projection from the AEC horizontal axis) while accessions G8 (Chijar) and G5 (Amarelo Cela) were the least stable (Fig. 4B).

### Correlations among traits

Pearson correlation coefficients indicated that seed yield was correlated to seed weight (r = 0.219; *P* value < 0.0001). Seed weight was positively correlated to water uptake (r = 0.271; *P* value < 0.0001) but negatively correlated to cooking time (r = −0.345; *P* value < 0.0001) (Table 5). Water uptake was negatively correlated with cooking time (r = −0.667; *P* value < 0.0001) implying that soaked beans that absorbed the most water cooked faster (Table 5).

**Table 5:** Pearson correlation coefficients among traits across the nine on-farm locations and two field seasons.

Traits	Yield	Seed weight	Water uptake	Cooking time
Yield	—	0.219*	−0.059 NS	−0.003 NS
Seed weight		—	0.271*	−0.345*
Water uptake			—	−0.667*

*Significant at the 0.0001 probability level; NS = not significant.

## Discussion

In Uganda, common bean is the most important legume as the crop can thrive in a wide range of agro-ecological zones^25^. Small seeded black Middle American common bean genotypes with indeterminate growth habits are productive in the drier parts of Northern Uganda such as Arua district. In the cooler mountainous regions of Southwestern Uganda farmers typically grow the Type IV large-seeded common bean varieties. These tend to be aggressive climbers and are often intercropped with maize for support^26^. The most widely grown and consumed beans in Uganda are Andean determinate types of the red mottled, yellow, and cream-colored market classes and these are grown across the Eastern, Central, and Western regions of Uganda^27,28^. The Andean determinate genotypes typically have a lower yield potential compared to the indeterminate Middle American accessions, but their shorter growing season and large seed sizes make them appealing to farmers and consumers^20^.

Five indeterminate genotypes were evaluated in this study along with the 12 determinate genotypes. The indeterminate genotypes were included since they had seed types within the scope of this study (i.e. red mottled and yellow). The two top yielding varieties were both indeterminate Middle American small-seeded genotypes. While these genotypes were all landraces with limited potential for direct release, their on-farm yield performance was similar to previous reports that showed indeterminate Middle American germplasm to produce higher yields and can thus be used as parents in breeding crosses to improve the determinate common bean types.

Both location and location by season components were important sources of variation for seed yield. Seed yield in common bean is a highly complex trait and affected by environmental factors^29,30^. There were also large differences in mean performance for yield across locations due to variability in disease pressure and soil characteristics with non-tropical varieties from North American being the most susceptible. Location TW in Kamuli district had insufficient soil NO_3_-N and lowest yield performance of 336 kg ha^−1^ demonstrating that soil fertility is a major challenge to common bean farmers in this region (Table 3; Supplementary Table S1).

The which-won-where GGE biplot is used to detect best performing genotypes in a group of environments or mega-environments for multi-environment field trials^31^. The polygon is generated by joining extreme genotypes in the study and perpendicular lines (rays) cutting through the sides of the polygon divide groups of genotypes and environments into sectors^32^. This study revealed presence of two mega environments for yield (Fig. 1A). Mega-environment identification has vital implications with regards to selection of test locations for genotype evaluation, selection, and release in modern breeding programs. Based on the results, the evaluation of genotypes for yield performance in Uganda should be conducted in two mega-environments.

Variability for seed weight in this study was largely controlled by genetics (51.5% of TSS). In common bean, seed size is strongly tied to gene pool structure. Andean genotypes are usually larger when compared to genotypes from the Middle America gene pool^33^. Most of the determinate genotypes were large kidney seed types weighing over 35 g per 100 seeds, which is a typical seed size among the large seeded Andean common bean^34^. Seed size along with cooking time and seed type are important end-use attributes valued by consumers in Uganda^15^. Identification of germplasm having large and stable seed size performance will help accelerate targeted development of common bean ideotypes that have traits important to farmers and consumers.

Hydration properties as measured by water uptake after 12-hour soaking, is an important parameter to evaluate seed quality in common bean. Typically, seeds will take up water at least 90% of their weight during a 12-hour soaking period. Hardshell is a phenomenon used to describe beans that take up insufficient water during soaking^35^. Hardshell is induced under semi-arid environmental conditions during seed fill and maturation and dry storage conditions^36^. Some genotypes are more susceptible to hardshell than others^37^. Hardshell is one reason for long cooking times, but it can often be reversed by storing harvested common bean seeds at ~68% relative humidity before cooking^38^. Genotypes G5 (Amarelo Cela), G8 (Chijar), and G9 (Vazon 7) that all belong to the Middle American gene pool were the most susceptible to the hardshell condition in this study. Genotypes grown at locations TW and GE in Kamuli district exhibited more hardshell defect as they imbibed the least amount of water.

Genotypes G5 (Amarelo Cela), G8 (Chijar), and G9 (Vazon 7) were also among the longest cooking accessions, showing that water uptake may be a useful predictor of long cooking time, although not always since G10 (PR0737-1) also had the second longest cooking time but still absorbed 110.7% water following a 12-hour soak. Therefore, genotype G10 (PR0737-1) likely has a different mechanism for long cooking time that is not related to the seed coat but could be related to its cotyledon structure. The longest cooking genotype G5 (Amarelo Cela) was a yellow grain colored landrace from Angola (Table 2). Interestingly, Katuuramu^39^ reported that genotype Amarelo Cela also accumulated the highest concentration of seed calcium. Presence of free calcium ions in the seed has been reported to form crosslinks with cell wall polysaccharides resulting in reduced cell separation and longer cooking times^40,41^.

The three fastest cooking genotypes G1 (Blanco Fanesquero), G2 (Ervilha), and G4 (Cebo Cela) cooked at least 10 minutes faster than the farmers’ local checks, indicating a potential valuable germplasm source that can be deployed to breed for fast cooking common bean for Uganda. The strong influence of genotype and the lack of large genotype by environment interactions for this cooking time suggest that breeding and evaluation for this trait can be carried out in fewer environments. In addition, the GGE biplot result showed presence of one mega-environment for cooking time (Fig. 2B) which further supports this recommendation.

The average environment coordinate (AEC) view (genotype mean performance vs. stability) allows for the comparison of genotypes based on estimates of genotype performance and stability across environments within a mega-environment^42^. Using this methodology, stability of genotypes is measured by the length of their projection from the AEC horizontal axis (shown by the dotted lines in Figs. 3 and 4). Ideally, genotypes with a near zero projection (e.g. absence of a dotted vertical line above or below the AEC horizontal axis) are declared stable^42^. Best performing genotypes for yield and seed weight were not stable across the nine on-farm locations (Fig. 3: Panels A and B). These findings show that genotypes responded differently to the existing soil nutrients, weather conditions, and endemic foliar disease pressures for each location, which further emphasize the strong influence of genotype by environment interactions on these agronomic traits. Projections from the AEC horizontal axes for the majority of the genotypes were shorter for water uptake and cooking time implying strong stability of these traits. For stability to be useful to plant breeders and growers, stable genotypes need to exhibit high trait performances and such results should be validated over multiple years of field evaluations^32^.

From a plant breeding perspective, correlation analysis can help identify positive or negative relationships among traits, identify novel parental combinations for variety development and detect trait measurement redundancy^43^. This study showed that yield was correlated to seed size. Previous studies have showed that increasing seed size results in a yield penalty and this has been one of the major limitations to yield improvements among genotypes in the Andean versus those in the Middle American gene pool^33^. No correlations were found between seed yield and water uptake or cooking time, suggesting that fast cooking varieties will not suffer a reduction in seed yield.

## Conclusions

Common bean genotypes with higher yields than the local checks were identified, although these had smaller seed size and longer cooking times than the farmers’ selected local checks. Fast cooking genotypes were also identified, including landraces G2 (Ervilha) and G4 (Cebo Cela), and the Ecuadorian white kidney variety G1 (Blanco Fanesquero) but none of the fastest cooking genotypes had higher yield than the preferred NABE-15 local check. There is a need to pyramid fast cooking and high yielding traits into a single genetic background and to evaluate preference of fast cooking genotypes among farmers and consumers. The moderate broad-sense heritability for on-farm seed yield and cooking time suggests phenotypic selection for these traits can allow for improvements in trait performances. With a single mega-environment for cooking time it is feasible that common bean germplasm evaluation and selection for this trait can be conducted in one or few test locations thus maximizing the value of resources available to the in-country breeding programs. On-farm varietal selection with farmers participation may be a well-suited strategy to identify fast cooking genotypes that appeal to growers and consumers.

## Materials and Methods

### Field study sites

The study was conducted at nine on-farm locations representing three agro-ecological zones across Hoima, Kamuli, Rakai, and Masaka districts in Uganda (Table 6). Hoima and Kamuli districts are located in Western and Eastern Uganda respectively. Both Rakai and Masaka districts are located in Central Uganda. These four districts were chosen because they represent areas of high common bean production, consumption, crop marketing, and selling. Both Kamuli and Hoima districts produce common bean largely for home consumption, while in Masaka and Rakai districts it is produced as both a food and cash crop^27^.

**Table 6:** Description of the nine on-farm locations used for the common bean genotype by environment study in Uganda.

District	Annual rainfall range (mm)	Annual temperature range (°C)	Agro-ecological zone	Location	Location code	Geographic coordinate		Altitude (m asl)	Soil type
Hoima	800–1,400	15–30	Grass land savanna	Kakindo	KA	N01°28.54ʹ	E031°25.46ʹ	1,228	Sandy clay
				Kyamalera	KY	N01°29.47ʹ	E031°26.99ʹ	1,174	Sandy clay loam
				Tugonzagane	TU	N01°16.93ʹ	E031°17.77ʹ	1,138	Clay
Kamuli	800–1,300	16–31	Tall savanna	Katugezeko	KU	N00°50.60ʹ	E033°12.11ʹ	1,127	Clay loam
				Tweweyo	TW	N00°54.79ʹ	E033°01.33ʹ	1,086	Sandy clay loam
				Tweyunge	GE	N00°53.77ʹ	E032°59.94ʹ	1,061	Sandy clay loam
Rakai	850–2,125	15–27	Tropical rain forest	Agali-awamu	AG	S00°34.87ʹ	E031°34.19ʹ	1,233	Sandy clay
				Kiyovu	KV	S00°43.58ʹ	E031°29.27ʹ	1,215	Sandy clay loam
Masaka	850–2,125	15–27	Tropical rain forest	Balitwewunya	BA	S00°25.54ʹ	E031°38.14ʹ	1,249	Clay

All study locations receive bimodal rainfall with the first rains falling from March to May while the second rainy season is from mid-September to early December. Hoima district receives rainfall ranging from 800 to 1,400 mm per year with temperature ranges of 15 to 30 °C^44^. Kamuli district receives an annual rainfall ranging from 800 to 1,300 mm with temperatures ranging from 16 to 31 °C^44^. Both Masaka and Rakai districts are located near Lake Victoria in Central Uganda and these districts receive an annual rainfall ranging from 850 to 2,125 mm. The annual temperature varies from 15 to 27 °C^44,45^. Across each of the on-farm locations variable amounts of rainfall, fluctuating number of rain days, and increasing changes in temperatures of 0.5 to 0.9 °C have been recently reported by Mubiru^44^.

Before planting, soil samples were collected from a soil layer depth of 0-to-30 cm at each of the nine on-farm sites for field seasons 2015 and 2016. The composite soil cores from each site were mixed and allowed to air-dry separately in Uganda, then shipped to the Soil and Plant Nutrient Laboratory at Michigan State University (http://www.spnl.msu.edu) for evaluation of nutrient composition. Soil fertility analysis was conducted to measure pH, organic matter (OM), nitrate-nitrogen (NO_3_-N), Bray-1 extractable phosphorus (P), potassium (K), and calcium (Ca) based on previously published protocols^46^.

The soils in Hoima district were sandy clay at KA, sandy clay loam at KY, and clay at TU. Soils in Kamuli district were clay loam for site KU, and sandy clay loam for both locations TW and GE. In Rakai district, AG had sandy clay while KV had sandy clay loam soils. Location BA in Masaka district had clay soils (Supplementary Table S1). The soils at each location had pH values ranging from 5.0 to 7.1 with locations in Rakai and Masaka districts (AG, KV, BA) having the most acidic soils with pH values ranging from 5.0 to 5.6 (Supplementary Table S1). One location in both Hoima (KY) and Kamuli (GE) had elevated soil pH levels over both years (Supplementary Table S1).

Most locations except TW (2016), AG (2015), and KV (2016) had organic matter greater or equal to 3% (Supplementary Table S1). Majority of the locations except KA and KY (both 2016), TW (2015 and 2016), and KV (2016) had sufficient soil NO_3_-N with readings above 11 µg g^−1^ which are considered optimum for common bean production. Soil P concentration ranged from 6–36 µg g^−1^ across all locations. Most sites except KY (2016) and BA (2015) had soil P levels below the optimum value of 36 µg g^−1^ required for good crop productivity on agricultural soils (Supplementary Table S1). Soil K concentrations ranged from 28–531 µg g^−1^ for the nine on-farm locations over the two field seasons. Soil K levels of 144 µg g^−1^ are sufficient for crop growth, and several locations did not meet this level of soil K for the 2015 and 2016 field seasons (Supplementary Table S1). In both years, soil Ca concentrations were not limiting at the nine on-farm locations with values above the 400 µg g^−1^ minimum cutoff that is desired for good crop performance (Supplementary Table S1).

### Common bean germplasm

The genotypes were chosen from a large Andean Diversity Panel (http://arsftfbean.uprm.edu/bean/) germplasm of over 200 lines previously field-grown in Montcalm, Michigan, USA in 2012 and 2013. The panel has been characterized for cooking time, seed mineral concentration, and iron bioavailability^20,47^. Genotypes targeted for inclusion in the 15-germplasm set had variable cooking times (from fast to slow cooking), iron (Fe) concentrations greater than 70 µg g^−1^ and zinc (Zn) concentrations greater than 30 µg g^−1^ on a dry weight basis^20,47^. Three of the genotypes were dark red kidney, four were yellow, five were red mottled, and there was one genotype from each of the white, small red, and light red kidney seed market classes. Most of the yellow and white colored genotypes were faster cooking and had higher Fe bioavailability when compared to the dark red kidney and red mottled accessions. The red mottled genotypes were particularly slower cooking but had higher concentrations of Fe and Zn^20,47^.

The local check NABE-15 (Kanyebwa) is a cream mottled common bean variety that is popular with farmers in Uganda and was grown at six locations (KA, TU, KY, GE, KV, BA) for field seasons 2015 and 2016. NABE-15 was released in 2010 by National Crops Resources Research Institute (NaCRRI) of Uganda with the attributes of high yield, fast cooking, and anthracnose resistance^48,49^. Locations KU and TW were planted with the Masindi yellow local check, which is an old landrace preferred for its taste and fast cooking attributes. The local check NABE-4 (Nambale omuwanvu) was grown in AG and was also released by NaCRRI in 1999. NABE-4 is a large red mottled variety described as fast cooking, drought tolerant, and high yielding^49^. Eleven genotypes and all local checks had type I determinate growth habit while the other five genotypes were indeterminate (Table 4).

### Field plot design

The experiment was conducted as a randomized complete block design with two field replications for all genotypes at the nine on-farm locations. Each genotype plot was comprised of five rows that were 3.5 m long with 0.5 m between row length. The entire five-row genotype plot was planted with 220 seeds. The study was conducted for two field seasons during the long rainy season of 2015 (September - through early December) and the shorter 2016 rainy season (March to May). Planting was initiated at the onset of rains (first week of September in 2015) and (first week of March in 2016). At planting, a light application of starter fertilizer containing nitrogen, phosphorus, and potassium (NPK: 17-17-17) was applied at a rate of 125 kg ha^−1^ to all sites in both field seasons. During each growing season the research plots were kept weed-free by two cycles of hand-weeding at 30- and 45-days post-planting. The trial plots were harvested and threshed by hand and all harvesting was completed by the first week of December in 2015 and third week of June in 2016.

### Phenotyping for common bean agronomic traits

Seed yield was collected on all harvested genotypes from a research plot area of 7 m^2^ and converted to kg ha^−1^. Seed weight was collected by counting 100 seeds and measuring their weight in grams.

### Phenotyping for hydration properties (water uptake) and cooking time

Prior to soaking and cooking, a total of 150 seeds for each genotype in both field seasons was placed in paper envelopes and kept at 4 °C (relative humidity of 75%) to ensure that seeds maintained a moisture content of 10–12%. Before conducting the water uptake and cooking time experiments, the seed moisture content of each genotype was measured using a Moisture Check Plus meter (Deere and Company, Moline, IL, USA). To measure the hydration properties (water uptake) of the common bean genotypes, 30 moisture equilibrated seeds were weighed and then soaked in distilled water (1:8 raw seed weight/water weight) for 12 hours at room temperature. The seeds were then drained, bloated dry, and reweighed to compute water uptake based on the following equation:

$${\rm{Water}}\,{\rm{uptake}}=\frac{{\rm{Seed}}\,{\rm{weight}}\,{\rm{after}}\,{\rm{soaking}}-{\rm{Seed}}\,{\rm{weight}}\,{\rm{before}}\,{\rm{soaking}}}{{\rm{Seed}}\,{\rm{weight}}\,{\rm{before}}\,{\rm{soaking}}}\times 100$$

To quantify cooking time, a total of 25 soaked seeds from each genotype were cooked using a pin-drop Mattson cooking device (Customized Machining and Hydraulics Co., Winnipeg, Canada). The Mattson cooker utilizes 2 mm stainless steel rods (each weighing 70 g) positioned above the center of each common bean seed during the cooking process. The Mattson cooker is then lowered into a four-liter stainless steel beaker containing 1.8 liters of boiling distilled water heated over a Waring SB30 portable burner. Cooking time was recorded as the number of minutes for taken for 80% of the pin-rods (i.e. 20 out of 25) to puncture through seeds under a steady boil at 100 °C^50^.

### Statistical analyses and data visualization

The analysis of variance (ANOVA) for all sources of variation was conducted using PROC MIXED statement using the statistical analysis software, SAS v9.4^51^. Pearson correlation coefficients among traits across locations and years were determined using the PROC CORR statement also in SAS. The variance components for estimating broad sense heritability (H^2^) for all traits were computed using the PROC VARCOMP statement in SAS v9.4 using the restricted maximum likelihood estimation method^51^. The statistical model used for the ANOVA and computing variance components in SAS v9.4 is shown below:

$$\begin{array}{ccc}{{\rm{Y}}}_{ijkm} & = & \mu +{{\rm{G}}}_{i}+{{\rm{L}}}_{j}+{{\rm{S}}}_{k}+{{\rm{GL}}}_{ij}+{{\rm{GS}}}_{ik}+{{\rm{LS}}}_{jk}\\  &  & +\,{{\rm{GLS}}}_{ijk}+{\rm{rep}}{({\rm{LS}})}_{jkm}+{\varepsilon }_{ijkm}\end{array}$$

where Y_*ijkm*_ is the response variable like yield or cooking time of the *i*^*th*^ common bean genotype in the *m*^*th*^ replication of the *j*^*th*^ location in the *k*^*th*^ season; µ is the grand mean; G_*i*_, L_*j*_, S_*k*_, GL_*ij*_, GS_*ik*_, LS_*jk*_, GLS_*ijk*_ are effects of the *i*^*th*^ genotype, *j*^*th*^ location, *k*^*th*^ season and their respective interactions; rep(LS)_*jkm*_ denotes the effect of the *m*^*th*^ replication nested within the interaction term of the *j*^*th*^ location and *k*^*th*^ season; ε_*ijkm*_ is the error term assumed to be normally distributed with mean = 0. For the ANOVA in SAS, the effects of G_*i*_, L_*j*_, and GL_*ij*_ were treated as fixed. The remaining effects were treated as random to estimate Fisher’s protected least significant difference (LSD) and to compare means among the common bean genotypes, as well as the locations for each phenotype. To generate the variance components for computing broad-sense heritability estimates (H^2^) all effects in the statistical model above were treated as random. Broad sense heritability (H^2^) was computed using the equation below:

$${\rm{Heritability}}=\frac{{\rm{Var}}({\rm{G}})}{{\rm{Var}}({\rm{G}})+\frac{{\rm{Var}}({\rm{GL}})}{{\rm{l}}}+\frac{{\rm{Var}}({\rm{GS}})}{{\rm{s}}}+\frac{{\rm{Var}}({\rm{GLS}})}{{\rm{ls}}}+\frac{{\rm{Var}}({\rm{Error}})}{{\rm{lsr}}}}$$

where, Var(G) is genotypic variance, Var(GL) is the genotype by location variance, Var(GS) is the genotype by season variance, Var(GLS) is the genotype by location by season variance, Var(Error) is the residual/experimental error variance. The denominators l, s, and r represent number of locations, seasons, and field replications respectively.

To visually assess the presence of mega-environments, trait stability, and genotype rankings^52^, a GGE biplot analysis was conducted using the GGEBiplotGUI package^53^ in RStudio. To generate two-way data required for GGE biplot analysis, all location by season combinations for each trait were defined as environments^32^ and the data files were inputted into RStudio to visualize the GGE patterns. All data were tester centered (G + GE) and non-scaled as described in Yan and Tinker^32^. Biplots intended to evaluate test environments and genotypes such as “which-won-where” polygons were drawn with column metric preserving singular value partitioning (SVP). Biplots for evaluating genotype and genotype by environment main effects, such as mean performance vs. stability and genotype rankings were drawn with row metric preserving SVP^54^.

## Supplementary information

Supplementary information S1.

## Data Availability

All the relevant data for this research are in the paper and its supplementary information files provided in the online version.
