# Supplementary material for: On-farm multi-location evaluation of genotype by environment interactions for seed yield and cooking time in common bean
Source: Sci Rep. 2020 Feb 27;10:3628. doi: 10.1038/s41598-020-60087-2 (PMC7046726; doi:10.1038/s41598-020-60087-2)
Supplement: Supplementary file 1 — Supplementary information S1. [file 41598_2020_60087_MOESM1_ESM.docx]

Electronic Supplementary Information for Scientific Reports Journal Article:

**On-farm multi-location evaluation of genotype by environment interactions for seed yield and cooking time in common bean**

Dennis N. Katuuramu^1^, Gabriel B. Luyima^2^, Stanley T. Nkalubo^2^, Jason A. Wiesinger ^1,3^, James D. Kelly^1^, & Karen A. Cichy^1,4^

^1^ Department of Plant, Soil and Microbial Sciences, Michigan State University, East Lansing, MI 48824, USA.

^2^ Legumes Research Program, National Crops Resources Research Institute, Namulonge, Kampala, Uganda.

^3^ USDA-ARS, Robert W. Holley Center for Agriculture and Health, Cornell University, Ithaca, NY, 14853, USA (current address)

^4^ USDA-ARS, Sugarbeet and Bean Research Unit, Michigan State University, East Lansing, MI, 48824, USA.

Correspondence and requests for materials should be addressed to K.A.C. (email: [karen.cichy@ usda.gov](mailto:karen.cichy@%20usda.gov))

Supplementary Table S1. Soil chemical composition analysis for the nine on-farm study locations over the two years in Uganda.

| District | Location | Year | Soil nutrient levels at planting | | | | | |
| --- | --- | --- | --- | --- | --- | --- | --- | --- |
|  |  |  | pH | OM (%) | NO_3_-N (µg g^-1^) | P (µg g^-1^) | K (µg g^-1^) | Ca (µg g^-1^) |
| Hoima | KA | 2015 | 6 | 6.7 | 21.2 | 12 | 144 | 1,998 |
|  |  | 2016 | 5.7 | 4.3 | 5.4 | 6 | 76 | 1,135 |
|  | KY | 2015 | 5.9 | 5.6 | 37.6 | 8 | 216 | 1,958 |
|  |  | 2016 | 6.3 | 3 | 4.4 | 36 | 45 | 1,482 |
|  | TU | 2015 | 6.2 | 3.7 | 22.9 | 10 | 195 | 1,283 |
|  |  | 2016 | 5.5 | 6.5 | 14.9 | 20 | 212 | 1,107 |
| Kamuli | KU | 2015 | 6.1 | 3 | 50.3 | 12 | 239 | 1,377 |
|  |  | 2016 | 5.7 | 4.6 | 16.3 | 13 | 83 | 1,158 |
|  | TW | 2015 | 6 | 4.8 | 8.3 | 13 | 94 | 1,509 |
|  |  | 2016 | 5.6 | 2.8 | 8.9 | 7 | 68 | 705 |
|  | GE | 2015 | 6 | 3.6 | 14.6 | 20 | 531 | 1,376 |
|  |  | 2016 | 7.1 | 3.5 | 11.9 | 23 | 305 | 2,038 |
| Rakai | AG | 2015 | 5 | 2.9 | 25.7 | 14 | 28 | 444 |
|  |  | 2016 | 5.5 | 4.3 | 11.8 | 23 | 83 | 1,019 |
|  | KV | 2015 | 5.4 | 4.2 | 13.5 | 20 | 63 | 951 |
|  |  | 2016 | 5.5 | 2.9 | 6.3 | 21 | 172 | 833 |
| Masaka | BA | 2015 | 5.2 | 4.6 | 22.9 | 36 | 171 | 1,290 |
|  |  | 2016 | 5.6 | 5.1 | 16.4 | 13 | 124 | 1,379 |

Supplementary Table S2. Genotype means for seed yield (kg ha^-1^) of the common bean accessions evaluated at the nine on-farm locations during the 2015 and 2016 field seasons in Uganda.

| Genotype code | Genotype name | Year | Location code | | | | | | | | |
| --- | --- | --- | --- | --- | --- | --- | --- | --- | --- | --- | --- |
|  |  |  | KA | KY | TU | KU | TW | GE | AG | KV | BA |
| G1 | Blanco Fanesquero | 2015 | 230 | 140 | 856 | 150 | 188 | 405 | 569 | 2108 | 275 |
|  |  | 2016 | 1125 | 1321 | 750 | 661 | 321 | 554 | 107 | 268 | 643 |
| G2 | Ervilha | 2015 | 247 | 492 | 766 | 265 | 184 | 571 | 416 | 1904 | 205 |
|  |  | 2016 | 1821 | 1393 | 1429 | 429 | 429 | 607 | 196 | 286 | 446 |
| G3 | PI527538 | 2015 | 488 | 248 | 972 | 483 | 169 | 746 | 446 | 1860 | 331 |
|  |  | 2016 | 1643 | 1571 | 1411 | 857 | 643 | 929 | 239 | 321 | 786 |
| G4 | Cebo Cela | 2015 | 359 | 90 | 534 | 278 | 186 | 426 | 631 | 946 | 380 |
|  |  | 2016 | 1357 | 1107 | 732 | 250 | 732 | 768 | 268 | 107 | 411 |
| G5 | Amarelo Cela | 2015 | 168 | 151 | 930 | 305 | 541 | 947 | 1143 | 3136 | 634 |
|  |  | 2016 | 2143 | 1286 | 1375 | 357 | 375 | 1036 | 589 | 286 | 982 |
| G6 | Maalasa | 2015 | 218 | 213 | 799 | 280 | 264 | 708 | 570 | 2081 | 507 |
|  |  | 2016 | 1268 | 1143 | 1196 | 214 | 304 | 839 | 150 | 204 | 339 |
| G7 | Rozi Koko | 2015 | 207 | 156 | 639 | 286 | 255 | 230 | 497 | 1791 | 245 |
|  |  | 2016 | 1357 | 1250 | 1661 | 321 | 500 | 482 | 196 | 179 | 357 |
| G8 | Chijar | 2015 | 266 | 559 | 1654 | 227 | 599 | 744 | 787 | 3216 | 1289 |
|  |  | 2016 | 1429 | 1982 | 1143 | 161 | 625 | 875 | 393 | 143 | 625 |
| G9 | Vazon 7 | 2015 | 211 | 166 | 1009 | 499 | 354 | 820 | 593 | 1102 | 710 |
|  |  | 2016 | 1571 | 857 | 1214 | 571 | 357 | 750 | 213 | 143 | 464 |
| G10 | PR0737-1 | 2015 | 651 | 257 | 966 | 353 | 319 | 759 | 734 | 2143 | 315 |
|  |  | 2016 | 1750 | 911 | 1214 | 357 | 179 | 661 | 232 | 232 | 732 |
| G11 | Kidungu | 2015 | 169 | 204 | 616 | 213 | 308 | 278 | 501 | 1631 | 551 |
|  |  | 2016 | 1661 | 1000 | 1071 | 161 | 250 | 393 | 429 | 214 | 446 |
| G12 | Uyole 96 | 2015 | 316 | 223 | 799 | 223 | 96 | 705 | 318 | 947 | 167 |
|  |  | 2016 | 1714 | 1196 | 714 | 643 | 143 | 286 | 125 | 357 | 482 |
| G13 | Charlevoix | 2015 | 176 | 241 | 689 | 218 | 156 | 841 | 412 | 1798 | 436 |
|  |  | 2016 | 1250 | 964 | 832 | 232 | 196 | 643 | 71 | 107 | 375 |
| G14 | Selian 97 | 2015 | 181 | 251 | 1331 | 480 | 174 | 500 | 787 | 1961 | 1112 |
|  |  | 2016 | 1518 | 1000 | 875 | 357 | 518 | 875 | 179 | 214 | 536 |
| G15 | Sacramento | 2015 | 154 | 150 | 521 | 407 | 304 | 714 | 504 | 1769 | 791 |
|  |  | 2016 | 1429 | 857 | 786 | 393 | 179 | 571 | 143 | 321 | 579 |
| G16 | NABE-15 | 2015 | 154 | 137 | 984 | - | - | 1081 | - | 1791 | 543 |
|  |  | 2016 | 1429 | 714 | 929 | - | - | 554 | - | 250 | 500 |
| Check-2 | NABE-4 | 2015 | - | - | - | - | - | - | 515 | - | - |
|  |  | 2016 | - | - | - | - | - | - | 429 | - | - |
| Check-3 | Masindi yellow | 2015 | - | - | - | 301 | 281 | - | - | - | - |
|  |  | 2016 | - | - | - | 268 | 625 | - | - | - | - |

Supplementary Table S3. Genotype means for seed weight (g) of the common bean accessions evaluated at the nine on-farm locations during the 2015 and 2016 field seasons in Uganda.

| Genotype code | Genotype name | Year | Location code | | | | | | | | |
| --- | --- | --- | --- | --- | --- | --- | --- | --- | --- | --- | --- |
|  |  |  | KA | KY | TU | KU | TW | GE | AG | KV | BA |
| G1 | Blanco Fanesquero | 2015 | 40.5 | 39.0 | 45.0 | 41.0 | 41.5 | 48.5 | 55.5 | 58.0 | 44.5 |
|  |  | 2016 | 50.4 | 48.9 | 45.0 | 40.2 | 46.0 | 46.1 | 39.4 | 41.6 | 43.9 |
| G2 | Ervilha | 2015 | 32.5 | 39.0 | 41.5 | 35.0 | 39.0 | 44.0 | 45.5 | 54.0 | 46.0 |
|  |  | 2016 | 54.3 | 40.7 | 39.3 | 42.8 | 42.2 | 42.3 | 39.1 | 38.7 | 42.6 |
| G3 | PI527538 | 2015 | 40.0 | 32.5 | 40.0 | 39.0 | 40.0 | 45.0 | 45.5 | 50.5 | 45.0 |
|  |  | 2016 | 44.6 | 39.3 | 37.2 | 38.0 | 42.5 | 40.4 | 31.6 | 32.9 | 37.8 |
| G4 | Cebo Cela | 2015 | 32.5 | 33.0 | 33.5 | 32.5 | 32.5 | 35.5 | 43.0 | 48.5 | 36.5 |
|  |  | 2016 | 36.3 | 30.3 | 31.4 | 30.2 | 43.7 | 33.7 | 34.2 | 29.3 | 36.6 |
| G5 | Amarelo Cela | 2015 | 24.5 | 22.0 | 26.5 | 21.5 | 26.0 | 28.0 | 34.5 | 35.0 | 28.0 |
|  |  | 2016 | 28.1 | 24.8 | 24.0 | 29.0 | 26.6 | 28.1 | 22.8 | 24.7 | 24.5 |
| G6 | Maalasa | 2015 | 37.0 | 31.5 | 40.0 | 33.5 | 42.0 | 42.5 | 40.0 | 56.5 | 39.5 |
|  |  | 2016 | 45.7 | 39.4 | 34.6 | 41.7 | 43.9 | 42.7 | 37.2 | 38.3 | 35.5 |
| G7 | Rozi Koko | 2015 | 40.5 | 34.5 | 41.0 | 39.5 | 40.0 | 42.0 | 48.0 | 58.5 | 45.0 |
|  |  | 2016 | 48.6 | 48.2 | 36.7 | 43.1 | 39.4 | 43.6 | 38.2 | 40.7 | 36.1 |
| G8 | Chijar | 2015 | 21.0 | 21.0 | 22.5 | 19.0 | 22.5 | 24.5 | 31.0 | 31.0 | 26.0 |
|  |  | 2016 | 27.2 | 24.8 | 24.2 | 26.9 | 25.5 | 28.0 | 24.2 | 24.1 | 26.4 |
| G9 | Vazon 7 | 2015 | 24.5 | 24.0 | 29.0 | 24.0 | 29.0 | 31.5 | 32.5 | 33.0 | 33.5 |
|  |  | 2016 | 35.0 | 28.8 | 25.7 | 33.7 | 34.1 | 36.8 | 27.9 | 32.6 | 31.1 |
| G10 | PR0737-1 | 2015 | 32.5 | 29.0 | 31.5 | 29.0 | 35.0 | 37.5 | 43.5 | 42.5 | 34.0 |
|  |  | 2016 | 39.3 | 32.5 | 27.7 | 37.6 | 30.6 | 42.4 | 25.4 | 34.4 | 31.7 |
| G11 | Kidungu | 2015 | 30.5 | 27.5 | 35.5 | 27.0 | 39.5 | 40.0 | 40.5 | 48.0 | 37.5 |
|  |  | 2016 | 37.6 | 32.9 | 30.8 | 33.3 | 34.0 | 25.9 | 34.9 | 27.1 | 31.7 |
| G12 | Uyole 96 | 2015 | 43.0 | 39.0 | 45.0 | 38.0 | 39.5 | 45.5 | 55.0 | 63.0 | 49.5 |
|  |  | 2016 | 53.1 | 45.7 | 38.2 | 40.4 | 39.8 | 41.1 | 40.3 | 45.7 | 42.8 |
| G13 | Charlevoix | 2015 | 35.0 | 38.0 | 43.5 | 36.5 | 41.5 | 42.5 | 47.5 | 52.5 | 44.0 |
|  |  | 2016 | 43.1 | 47.3 | 42.3 | 38.9 | 41.9 | 42.9 | 38.9 | 36.1 | 36.8 |
| G14 | Selian 97 | 2015 | 31.5 | 30.0 | 34.5 | 33.0 | 39.0 | 32.5 | 46.5 | 49.0 | 43.5 |
|  |  | 2016 | 38.2 | 32.0 | 30.3 | 35.7 | 31.3 | 33.1 | 33.5 | 28.7 | 31.1 |
| G15 | Sacramento | 2015 | 35.0 | 32.0 | 42.5 | 35.5 | 38.5 | 39.0 | 56.0 | 53.5 | 43.5 |
|  |  | 2016 | 43.8 | 42.5 | 40.1 | 38.7 | 39.3 | 37.0 | 31.3 | 39.1 | 38.7 |
| G16 | NABE-15 | 2015 | 35.5 | 29.5 | 43.0 | - | - | 45.0 | - | 51.5 | 51.0 |
|  |  | 2016 | 40.4 | 41.5 | 32.3 | - | - | 40.4 | - | 30.1 | 37.0 |
| Check-2 | NABE-4 | 2015 | - | - | - | - | - | - | 53.5 | - | - |
|  |  | 2016 | - | - | - | - | - | - | 36.0 | - | - |
| Check-3 | Masindi yellow | 2015 | - | - | - | 35.0 | 45.5 | - | - | - | - |
|  |  | 2016 | - | - | - | 44.6 | 33.9 | - | - | - | - |

Supplementary Table S4. Genotype means for water uptake (%) of the common bean accessions evaluated at the nine on-farm locations during the 2015 and 2016 field seasons in Uganda.

| Genotype code | Genotype name | Year | Location code | | | | | | | | |
| --- | --- | --- | --- | --- | --- | --- | --- | --- | --- | --- | --- |
|  |  |  | KA | KY | TU | KU | TW | GE | AG | KV | BA |
| G1 | Blanco Fanesquero | 2015 | 123.1 | 114.3 | 110.9 | 111.6 | 112.0 | 117.1 | 102.8 | 107.8 | 118.8 |
|  |  | 2016 | 117.1 | 112.6 | 108.8 | 107.5 | 106.2 | 99.5 | 118.9 | 111.6 | 106.4 |
| G2 | Ervilha | 2015 | 136.4 | 126.5 | 131.9 | 129.4 | 131.8 | 125.8 | 112.3 | 108.8 | 118.6 |
|  |  | 2016 | 125.5 | 118.3 | 111.9 | 113.7 | 107.6 | 104.8 | 119.7 | 116.8 | 108.1 |
| G3 | PI527538 | 2015 | 109.7 | 115.8 | 123.0 | 114.3 | 117.1 | 106.9 | 105.1 | 101.8 | 106.2 |
|  |  | 2016 | 110.7 | 107.4 | 108.2 | 96.5 | 93.2 | 92.9 | 108.4 | 108.0 | 97.5 |
| G4 | Cebo Cela | 2015 | 122.0 | 118.6 | 118.3 | 117.5 | 122.1 | 105.1 | 104.9 | 108.6 | 115.6 |
|  |  | 2016 | 127.6 | 118.7 | 115.6 | 114.6 | 105.0 | 103.8 | 119.9 | 117.7 | 107.6 |
| G5 | Amarelo Cela | 2015 | 23.3 | 41.1 | 18.7 | 39.8 | 20.0 | 35.6 | 70.1 | 64.5 | 82.7 |
|  |  | 2016 | 93.3 | 107.9 | 101.8 | 97.8 | 90.0 | 86.6 | 102.4 | 103.9 | 101.8 |
| G6 | Maalasa | 2015 | 116.8 | 112.1 | 109.5 | 113.1 | 114.2 | 120.9 | 101.5 | 103.2 | 112.3 |
|  |  | 2016 | 126.3 | 118.9 | 114.9 | 116.7 | 105.7 | 104.6 | 119.9 | 111.2 | 112.3 |
| G7 | Rozi Koko | 2015 | 122.7 | 119.9 | 116.3 | 116.9 | 115.3 | 121.7 | 106.9 | 108.0 | 120.2 |
|  |  | 2016 | 122.9 | 115.0 | 119.5 | 107.1 | 109.6 | 109.3 | 112.1 | 112.7 | 113.2 |
| G8 | Chijar | 2015 | 92.9 | 94.7 | 102.1 | 64.3 | 54.7 | 114.1 | 96.0 | 102.0 | 104.4 |
|  |  | 2016 | 119.5 | 108.4 | 105.8 | 108.5 | 100.6 | 95.2 | 114.7 | 111.8 | 107.4 |
| G9 | Vazon 7 | 2015 | 63.3 | 34.0 | 67.5 | 61.7 | 76.7 | 79.0 | 82.6 | 80.5 | 89.8 |
|  |  | 2016 | 116.7 | 110.0 | 122.1 | 105.7 | 104.5 | 98.9 | 113.3 | 112.4 | 109.4 |
| G10 | PR0737-1 | 2015 | 122.0 | 116.6 | 116.2 | 116.5 | 113.3 | 113.4 | 107.9 | 110.0 | 120.3 |
|  |  | 2016 | 116.5 | 118.8 | 116.2 | 102.9 | 103.8 | 90.5 | 105.7 | 102.7 | 99.4 |
| G11 | Kidungu | 2015 | 101.4 | 103.7 | 106.3 | 108.9 | 113.5 | 102.3 | 100.6 | 91.0 | 94.1 |
|  |  | 2016 | 106.2 | 101.3 | 102.4 | 92.8 | 92.1 | 93.9 | 100.6 | 111.1 | 98.4 |
| G12 | Uyole 96 | 2015 | 109.1 | 111.6 | 113.6 | 115.8 | 114.9 | 118.4 | 103.1 | 98.8 | 97.6 |
|  |  | 2016 | 111.4 | 108.2 | 110.2 | 105.6 | 100.0 | 96.8 | 107.8 | 109.8 | 99.1 |
| G13 | Charlevoix | 2015 | 126.3 | 127.6 | 126.7 | 132.8 | 129.0 | 127.7 | 115.5 | 112.6 | 121.6 |
|  |  | 2016 | 135.8 | 118.2 | 114.1 | 115.3 | 117.0 | 103.5 | 122.0 | 127.2 | 124.2 |
| G14 | Selian 97 | 2015 | 111.4 | 122.5 | 121.6 | 119.7 | 105.1 | 119.9 | 109.8 | 104.4 | 105.8 |
|  |  | 2016 | 121.7 | 121.8 | 121.5 | 107.8 | 108.7 | 102.8 | 117.0 | 118.5 | 111.2 |
| G15 | Sacramento | 2015 | 116.5 | 127.6 | 117.9 | 119.9 | 121.0 | 115.3 | 113.3 | 102.5 | 110.2 |
|  |  | 2016 | 118.2 | 116.8 | 108.4 | 102.4 | 105.7 | 100.8 | 120.2 | 111.6 | 107.8 |
| G16 | NABE-15 | 2015 | 102.2 | 149.1 | 109.0 | - | - | 110.1 | - | 95.5 | 96.4 |
|  |  | 2016 | 112.9 | 104.8 | 102.5 | - | - | 92.5 | - | 110.6 | 103.3 |
| Check-2 | NABE-4 | 2015 | - | - | - | - | - | - | 96.1 | - | - |
|  |  | 2016 | - | - | - | - | - | - | 121.1 | - | - |
| Check-3 | Masindi yellow | 2015 | - | - | - | 113.7 | 112.1 | - | - | - | - |
|  |  | 2016 | - | - | - | 95.5 | 95.4 | - | - | - | - |

Supplementary Table S5. Genotype means for cooking time (min) of the common bean accessions evaluated at the nine on-farm locations during the 2015 and 2016 field seasons in Uganda.

| Genotype code | Genotype name | Year | Location code | | | | | | | | |
| --- | --- | --- | --- | --- | --- | --- | --- | --- | --- | --- | --- |
|  |  |  | KA | KY | TU | KU | TW | GE | AG | KV | BA |
| G1 | Blanco Fanesquero | 2015 | 28.0 | 29.2 | 24.5 | 25.5 | 27.8 | 20.1 | 25.4 | 26.7 | 26.0 |
|  |  | 2016 | 29.2 | 36.1 | 28.1 | 22.3 | 35.4 | 35.9 | 26.9 | 30.0 | 30.6 |
| G2 | Ervilha | 2015 | 28.1 | 25.7 | 22.9 | 25.3 | 25.9 | 27.9 | 29.6 | 23.7 | 27.2 |
|  |  | 2016 | 25.1 | 31.4 | 28.2 | 21.2 | 36.2 | 31.0 | 24.3 | 25.5 | 27.0 |
| G3 | PI527538 | 2015 | 53.9 | 39.9 | 32.5 | 39.3 | 53.0 | 43.5 | 44.9 | 52.1 | 53.8 |
|  |  | 2016 | 36.4 | 44.2 | 42.3 | 33.2 | 61.5 | 46.2 | 37.6 | 35.2 | 41.6 |
| G4 | Cebo Cela | 2015 | 21.3 | 22.4 | 21.2 | 20.7 | 28.5 | 23.7 | 27.2 | 26.0 | 25.1 |
|  |  | 2016 | 21.7 | 26.1 | 25.1 | 21.3 | 36.4 | 26.8 | 25.6 | 19.4 | 23.9 |
| G5 | Amarelo Cela | 2015 | 124.5 | 121.7 | 145.1 | 123.0 | 270.6 | 114.4 | 82.4 | 93.7 | 60.9 |
|  |  | 2016 | 45.4 | 96.5 | 99.4 | 46.1 | 105.0 | 53.3 | 51.6 | 50.7 | 52.8 |
| G6 | Maalasa | 2015 | 35.5 | 30.7 | 35.2 | 31.0 | 42.1 | 38.7 | 62.6 | 36.6 | 35.7 |
|  |  | 2016 | 26.1 | 29.2 | 25.1 | 23.9 | 40.5 | 28.5 | 31.9 | 30.2 | 59.9 |
| G7 | Rozi Koko | 2015 | 36.8 | 36.6 | 28.1 | 34.6 | 48.2 | 28.4 | 32.5 | 32.0 | 36.6 |
|  |  | 2016 | 29.2 | 28.7 | 30.9 | 24.6 | 39.9 | 28.5 | 30.2 | 28.9 | 34.8 |
| G8 | Chijar | 2015 | 66.7 | 53.6 | 35.2 | 77.9 | 254.5 | 38.8 | 33.1 | 31.4 | 39.8 |
|  |  | 2016 | 29.3 | 59.9 | 35.3 | 31.9 | 52.5 | 33.7 | 35.1 | 29.2 | 31.1 |
| G9 | Vazon 7 | 2015 | 106.6 | 126.0 | 131.3 | 68.3 | 95.1 | 84.6 | 80.4 | 71.2 | 46.1 |
|  |  | 2016 | 32.9 | 53.7 | 42.7 | 35.0 | 49.2 | 32.8 | 49.6 | 29.2 | 34.5 |
| G10 | PR0737-1 | 2015 | 62.7 | 59.4 | 74.1 | 54.0 | 83.3 | 61.6 | 66.5 | 52.4 | 63.3 |
|  |  | 2016 | 50.3 | 125.4 | 74.1 | 57.5 | 109.5 | 57.3 | 99.9 | 65.6 | 85.7 |
| G11 | Kidungu | 2015 | 43.3 | 57.9 | 43.9 | 30.7 | 41.5 | 48.9 | 64.9 | 48.3 | 42.5 |
|  |  | 2016 | 37.7 | 56.0 | 34.9 | 34.5 | 70.5 | 42.6 | 35.1 | 34.0 | 38.7 |
| G12 | Uyole 96 | 2015 | 44.3 | 47.5 | 35.8 | 33.5 | 46.2 | 33.2 | 75.0 | 39.9 | 36.9 |
|  |  | 2016 | 34.2 | 50.5 | 39.2 | 22.6 | 48.7 | 46.8 | 39.7 | 29.6 | 39.1 |
| G13 | Charlevoix | 2015 | 57.1 | 54.6 | 38.7 | 48.9 | 56.4 | 57.1 | 66.1 | 65.9 | 64.9 |
|  |  | 2016 | 35.3 | 40.0 | 38.6 | 46.6 | 60.9 | 40.0 | 33.6 | 37.6 | 38.4 |
| G14 | Selian 97 | 2015 | 32.6 | 32.0 | 35.1 | 30.6 | 42.4 | 26.9 | 44.5 | 31.0 | 35.5 |
|  |  | 2016 | 28.2 | 33.3 | 32.6 | 25.4 | 45.0 | 33.4 | 24.2 | 29.8 | 33.0 |
| G15 | Sacramento | 2015 | 56.2 | 47.8 | 32.7 | 47.4 | 59.7 | 45.9 | 43.7 | 47.8 | 44.9 |
|  |  | 2016 | 41.5 | 40.9 | 38.5 | 24.0 | 42.1 | 46.9 | 37.0 | 40.5 | 37.6 |
| G16 | NABE-15 | 2015 | 35.9 | 40.1 | 33.3 | - | - | 44.9 | - | 38.2 | 36.2 |
|  |  | 2016 | 39.9 | 43.0 | 29.4 | - | - | 35.9 | - | 31.3 | 30.3 |
| Check-2 | NABE-4 | 2015 | - | - | - | - | - | - | 45.2 | - | - |
|  |  | 2016 | - | - | - | - | - | - | 31.2 | - | - |
| Check-3 | Masindi yellow | 2015 | - | - | - | 39.8 | 41.0 | - | - | - | - |
|  |  | 2016 | - | - | - | 33.4 | 58.4 | - | - | - | - |
